# Study on Transformation of Ginsenosides in Different Methods

**DOI:** 10.1155/2017/8601027

**Published:** 2017-12-14

**Authors:** Meng-meng Zheng, Fang-xue Xu, Yu-juan Li, Xiao-zhi Xi, Xiao-wei Cui, Chun-chao Han, Xue-lan Zhang

**Affiliations:** School of Pharmacy, Shandong University of Traditional Chinese Medicine, Jinan 250355, China

## Abstract

Ginseng is a traditional Chinese medicine and has the extensive pharmacological activity. Ginsenosides are the major constituent in ginseng and have the unique biological activity and medicinal value. Ginsenosides have the good effects on antitumor, anti-inflammatory, antioxidative and inhibition of the cell apoptosis. Studies have showed that the major ginsenosides could be converted into rare ginsenosides, which played a significant role in exerting pharmacological activity. However, the contents of some rare ginsenosides are very little. So it is very important to find the effective way to translate the main ginsenosides to rare ginsenosides. In order to provide the theoretical foundation for the transformation of ginsenoside in vitro, in this paper, many methods of the transformation of ginsenoside were summarized, mainly including physical methods, chemical methods, and biotransformation methods.

## 1. Introduction

Ginseng (Panax ginseng Meyer) is a traditional Chinese medicine, which is used in ancient eastern countries. Ginseng is used as energy booster and can fortify the spleen to benefit the lungs, nourish fluids, calm the heart, tranquilize the mind, and so on [1–3]. Ginsenosides and polysaccharides are the main active substances in ginseng. Studies on the ginseng polysaccharides are few [4–6]. But there are a lot of reports about ginsenosides. Ginseng has so many pharmacological activities because of ginsenosides. According to the difference in the position and quantity of sugar moiety in the glycosides, ginsenosides are divided into three types: protopanaxadiol (PPD), protopanaxatriol (PPT), and oleanolic acid [7, 8]. At present, 60 ginsenosides approximately were isolated and identified from family Araliaceae. Ginsenosides showed many bioactive and pharmacological activities, such as anticancer, anti-inflammatory, antiaging, and antioxidant [9–12]. In addition, some researchers suggested that minor ginsenoside and aglycone are superior to the major ginsenoside at antitumor [13]. The antitumor activity is decreasing with the increasing sugar moiety in the glycosides [14]. But the contents of minor ginsenoside and aglycone are low or few in ginseng. Minor ginsenosides extracted from plants cannot satisfy the scientific researches and the clinical requirements. So it is very significant to prepare the minor ginsenoside through transforming the major ginsenoside. In recent years, the researches about the conversion of ginsenosides have obtained many significant results and studies on the transformation mechanism also made some progress [15, 16].

**Figure 1:**
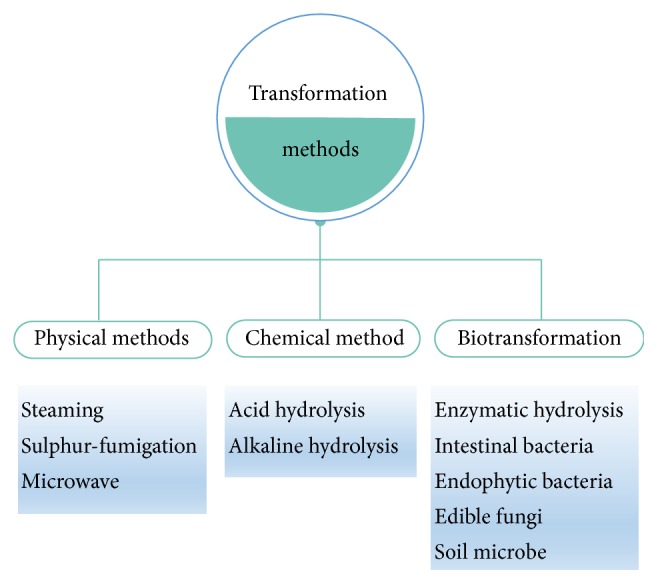
Methods applied on the transformation of ginsenosides. Three types of methods were concluded, which included the physical methods, chemical method, and biotransformation. Each type of method contained several kinds of common method that transformed a ginsenoside into another kind of ginsenoside by changing ginsenoside's chemical structure.

In this paper, we summarized many methods of the transformation of ginsenosides, mainly including physical methods, chemical methods, and biotransformation (Figure 1). The purpose of this study is to provide the theoretical basis for the new methods and approaches for the transformation and biosynthesis of ginsenosides.

## 2. Physical Methods

### 2.1. Steaming Transformation

Heat-treatment of ginseng is a conventional way that is widely applied in ancient times. Ginseng could product some rare ginsenosides when heated at a higher temperature than conventional preparation. To study on the transformation induced by steaming, red ginseng samples were prepared by steaming of white ginseng at 98°C for 3 h or 1 h and drying at 60°C for 12 h. The results proved that some malonyl ginsenosides were generated in the heated white ginseng, while they were not detected in the steaming ginseng, and the contents of malonyl ginsenosides would be decreased during steaming. This change might be related to enzymes which convert malonyl ginsenosides to corresponding neutral ginsenosides [17]. Chemical constituents and biological activities of ginseng would be changed when ginseng was steamed at a temperature over 100°C. After steaming at 120°C, some ginsenosides were produced, such as ginsenoside Rg3, F4, and Rg5, which did not exist at raw ginseng. But the contents of ginsenoside Rf, Re, Rd, Rc, Rb1, and Rb2 were decreased after steaming and were fewer when steamed at 120°C [18]. Besides, when ginseng steamed at 120°C for 3 h, the contents of some ginsenosides in the methanol extract were showed as follows: 20(S)-Rg3 (0.700%), 20(R)-Rg3 (0.643%), Rg5 (0.492%), and other saponins (1.37%) [19]. In addition, an autoclave was used to steam the root of fresh ginseng, and HPLC was used to defected the change of ginseng's constituents. On the one hand, temperature has a significant influence on the conversion of ginsenosides. For example, the contents of total ginsenosides were increased after ginseng roots were steamed at 98°C and decreased gradually at 120°C. On the other hand, time was also important. For instance, the contents of Rb1, Rb2, Rc, and Rd were increased sharply within first 0.5 h at 120°C and almost disappeared after 4 h. Moreover, acidic ginsenosides and neutral ginsenosides would be converted to rare ginsenosides during the root steaming. Acidic ginsenosides play an important role in the transformation of ginsenosides because they can release malonic acid and acetic acid, which affect the process of the transformation of saponins [20]. What is more, the combination of resin adsorption and heating may have a better application in conversion of ginsenosides. Total ginsenosides were obtained by 70% (v/v) ethanol and loaded to HP-20 open column. The ginsenosides enriched fractions were eluted by 65% methanol. The extraction from HP-20 was heated in autoclave at 130°C for 4 h, and the steam-treated extraction was separated by AB-8 and identified using HPLC-APLC-MS. The result showed that polar ginsenosides can convert to less polar ones, and PPD group could translate into 20(S)/(R)-Rg3 by selectively eliminating the C-20 sugar chain at high temperature and pressure. The transformed pathways of some ginsenosides by heat were as follows: Rb1, Rb2, Rc, Rd → Rg3 → Rk1, Rg5, Rs3; Re → Rg2 → Rg6 and F4; Rg1 → Rh1 [21].

### 2.2. Sulphur-Fumigation Transformation

Not only sunlight or heating is traditional method to process ginseng, but also sulphur-fumigation is also a convenient way to handle ginseng and other medicinal herbs. The slices of ginseng and sulphur powder were put into a desiccator at the lower and upper layer at 25°C for 12 h, respectively. The sulphur-fumigated ginseng was extracted by 75% ethanol at 85°C for 3 h. The experimental results suggested that some new compounds appeared in sulphur-fumigated ginseng comparing with reference compounds and nonfumigated ginseng by using UPLC-QTOF-MS/MS, such as ginsenosides Rh2 and Rg5 [22]. Some ginsenosides can be converted to ginsenoside derivatives or sulfate in sulphur-fumigation process through hydrolysis, dehydration, and decarboxylation [22–24]. Furthermore, sulphur-fumigation can influence the contents of original ginsenosides. For example, the contents of Rg1, Re, Rc, Rb2, and Rd were all decreased after sulphur-fumigation. The total contents of 10 ginsenosides in sulphur-fumigated white ginseng were decreased by up to 64% comparing with nonfumigated white ginseng [22]. Sulphur-fumigated white ginseng from different country could be discriminated by the chemical fingerprint of sulphur-fumigated ginseng. This also provides the useful way to identify other sulphur-fumigated medicinal herbs [23].

### 2.3. Microwave Transformation

The traditional processing methods of ginseng include conventional heating and sulphur-fumigation, but they have some drawbacks. The heating process is slow and the sulphur-fumigation would produce some hazardous substance. Recently, microwave heating has been applied on natural products and microwave heating is a simple, efficient, and time saving way to process products. The floatation solution of ginseng was put into the microwave irradiation system and the degradation of ginsenosides was continuously performed for 5 min under 165°C. The solutions after degradation were extracted by water-saturated n-butanol and evaporated to dryness. The results showed that ginsenosides Rh2 and Rg3 were the only degradation product of ginsenoside Rc and Rd, respectively. The yields of ginsenoside Rg3, Rh2 and aglycone are the highest under 165°C and 15 min. The highest transformation rate of total ginsenosides to ginsenoside Rg3 was 47.98% in neutral solution. And the conversion rate of major ginsenosides to aglycon was 78.11% in alkaline solution. Moreover, the yield of ginsenoside Rg3 obtained by microwave is 7.69 mg/g and 250 times as high as that obtained from red ginseng. The mechanism of saponins conversion may be related to hydrolyze glycosidic bond at C-20 and C-3 [25]. In addition, the degradation of ginsenosides may follow a first-order reaction in ethanol solution. And neutral ginsenosides are more stable than malonyl ginsenosides. The values of the rate constants of ginsenosides degradation were similar at the same temperature in both aqueous and 50% ethanol-water extracts between the microwave and conventional heating methods. So microwave could replace conventional heating methods to apply on the extraction of ginsenosides [26].

## 3. Chemical Methods

### 3.1. Acid Hydrolysis

**Figure 2:**
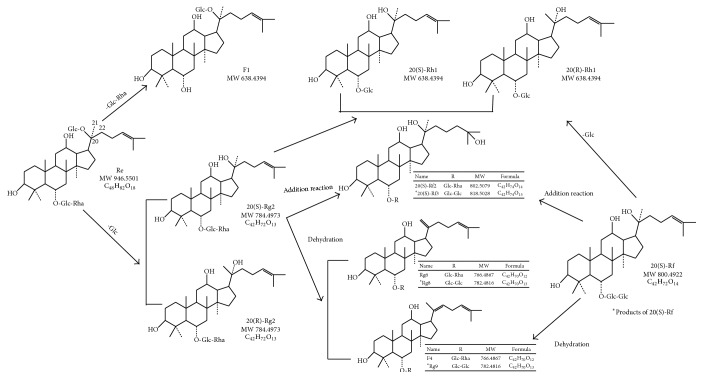
Data redrawn from [29]. Chemical transformation pathways of 20(S)-PPT ginsenosides Re, Rg2, and Rf. The transformation mechanisms of 20(S)-PPT ginsenosides Re, Rg2, and Rf include hydrolysis of saccharide substitution at C-6, Δ20(21) or Δ20(22) dehydration, and hydration addition reactions at C-24 and C-25.

As we all know, ginsenosides can be decomposed to corresponding prosapogenins under acidic condition. Major ginsenosides could produce different rare ginsenosides under different acidic conditions. Ginsenosides Rg1 and Re were treated with 0.1 N-HCL at 37°C for 120 min. N-BuOH extracts of the mixtures were applied to silica gel column chromatography to get the prosapogenin of Rg1 and Re. Rg1-prosapogenin I and Re-prosapogenin I were not detected because they were decomposed to other complicated mixtures [27]. What is more, the pH is a determinant factor in the transformation process of ginsenosides. Ginsenosides could not be transformed under the neutral condition and would produce by-products under strong acid. Different concentrations of formic acid (0.01%, 0.1%, 0.5%, 2%, and 5%) were applied to the conversion of some sapiens. The results showed that 0.01% of formic acid provided the highest yields. Ginsenosides Rh1, Rh2, and Rg3, with 0.01% formic acid at 120°C for 4 h, could transform into ginsenosides Rk3 and Rh4, Rk1 and Rg5, and Rk2 and Rh3, respectively. Besides, the total transformation efficiency was more than 20% through formic acid-treating. And the cytotoxic effect of ginsenosides on human cancer cells is inversely related to the sugar number and sugar linkages rank as C-3 > C-6 > C-20 [28]. 20(S)-Protopanaxatriol- (PPT-) type ginsenosides standards Re, Rg2, and Rf were dissolved by 50% (v/v) methanol/water and the liquor PH was adjusted to 2.0 with formic acid. The liquors of saponins were heated to reflux (60°C) for 120 mins. The result demonstrated that 20(S)-Re could produce eight compounds; they were 20(S)-Rf2, 20(S)-Rg2, 20(R)-Rg2, 20(S)-Rh1, 20(R)-Rh1, F1, Rg6, and Rg4. 20(S)-Rg2 produced five compounds; they were 20(S)-Rf2, 20(S)-Rh1, 20(R)-Rh1, Rg6, and Rg4. And 20(S)-Rf produced five compounds; they were 20(S)-Rh1, 20(R)-Rh1, 20(S)-Rf3, Rg8, and Rg9. The transformation mechanisms of 20(S)-protopanaxatriol- (PPT-) type ginsenosides include hydrolysis of saccharide substitution, Δ20(21) or Δ20(22) dehydration, and hydration addition reactions at C-24 and C-25. The chemical transformation pathways of 20(S)-PPT ginsenosides Re, Rg2, and Rf were showed in Figure 2 [29]. Beside formic acid, these acids can also be used in chemical transformation of ginsenosides, such as acetic acid, citric acid, lactic acid, tartaric acid, and HCL. Protopanaxadiol (PPD) ginsenosides could transform into Rg3 and Δ^20^-ginsenoside Rg3 under acid conditions. The yield of Rg3 was increased by increasing incubation temperature and time in acidic condition. The optimal time and temperature of transformation were 5 h and 60°C, respectively. What is more, ginsenosides Rb1, Rb2, and Rc were incubation at 60°C in acetic acid, citric acid, lactic acid, tartaric acid, and HCL and the levels of transformed ginsenosides were measured. Compared with lactic acid and citric acid, HCL could increase the yield of 20(R)-ginsenoside Rg3 and Δ^20^-ginsenoside Rg3 to 20(S)-ginsenoside Rg3. The transformation of ginsenosides by 0.1% acids was better than that of 1% acids. But the transformation of ginsenosides to Δ^20^-ginsenoside Rg3 by 0.1% acid was not greater than that by 1% acids. The results demonstrated that ginsenosides were easily transformed in acidic conditions. However, it is important to control the PH, time, and temperature for the conversion of ginsenosides in acid condition [30].

### 3.2. Alkaline Hydrolysis

Alkaline hydrolysis is a method to decompose saponins on the condition of high temperature, high pressure, and alkaline to obtain secondary saponins. But alkaline hydrolysis also has a strict requirement on the reaction temperature, pressure, and PH. Total saponins would be hydrolyzed in boiling water bath with 2 mol/L NaOH aqueous solution for 8 h and extracted by EtoAc. It is interesting to note that a new compound, 20(R)-ginsenoside Rh19, could be isolated and identified by NMR and MS spectra analyses [31]. For alkaline hydrolysis, temperature and PH are two important conditions. In general, at the same time, the higher the PH, the faster the hydrolysis. The hydrolysis rate of ginsenoside Ro was also related to temperature and PH. The hydrolysis rate changes more obviously on the condition of PH 13 and 60°C, mainly hydrolyzing the ester bond at C-28. The hydrolysate of ginsenoside Ro by alkali was zingibroside-R1 [32]. Moreover, the yield of protopanaxadiol was the highest by alkaline hydrolysis when the solvent is isoamyl alcohol, and hydrolysis time is at 24 h, normal pressure. But the yield of protopanaxadiol would be decreased when the heating time is more than 32 h in isoamyl alcohol. This showed that prolonged heating can lead to the degradation of saponins in high temperatures [33].

These chemical methods also have some disadvantages, including poorly selective, epimerization, hydration, hydroxylation, and environmental pollution [34].

## 4. Biotransformation of Ginsenosides

Biotransformation is a way which makes use of organisms or enzyme as catalyst to realize the process of chemical conversion and modify the structure of the external substrate. The advantages of biotransformation are strongly selective, mildly reaction conditions, less by-products, simple reprocessing, friendly environment, and so on. The biotransformation of ginsenosides is mainly using microorganism or enzyme to decorate the glucosyl of ginsenosides at C-3, C-6, and C-20 to translate rare saponins. The main methods of biotransformation are enzyme conversion and microbial transformation.

### 4.1. Enzymatic Hydrolysis

The enzymatic way has been deemed to a promising method to generate rare ginsenosides, owing to its short reaction cycle, little pollution, high purity, and high specificity [34]. Different enzymes play a different role in hydrolyzing ginsenosides. Rapidase, Econase CE, Viscozyme, Ultraflo L, and Cytolase PC15 were used for secondary enzymatic hydrolysis after amylase treatment of ginseng extract. The hydrolysis reaction was stopped by boiling for 15 min. The results showed that Rapidase can not only significantly increase the total contents of ginsenosides, but also increase the contents of panaxadiols and panaxatriols, such as Rh1, Rg5, Rk1, Rg2, and Rg3. However, the effect of Ultraflo L is opposite to Rapidase. Rc was the most ample in the control, but the contents of Rc are decreased after being treated with various enzymes. Some researches indicated that the hydrolysis of carbohydrates enhances the extraction of ginsenosides and shorter sugars [35]. Some commercial enzymes, Cytolase PCL5, Sumizyme AC, Multifect Pectinase FE, and Crystalzyme PML-MX, were added to the ginseng extract, mainly including ginsenosides Rb1, Rc, Rb2, and Rd. After 60 h of treatment by enzyme, these major ginsenosides were transformed into Rg3, F2 and compound K. what is more, the effects of Cytolase PCL5 and Sumizyme AC are better than other enzymes at conversion of ginsenosides. And Cytolase PCL5 is superior to Sumizyme AC because the yield of compound K is increased as the temperature decreased. The optimal conditions were identified as 78 h of treatment at PH 4.3 at 55.4°C. This result could provide a practical basis for other methods to hydrolyze saponins [36]. As is well known, ginsenoside Rg3 has the good effect on anticardiovascular and antimetastasis. But ginsenoside Rg3 is not found in the white ginseng and the content of it is very low in red ginseng. The 20(S)-Rg3 isomer is more water-soluble and more bioavailable than the 20(R)-Rg3 isomer. There are few reports about the conversion of ginsenoside Rg3 by the enzymes. So it is important to explore the optimal condition for the transformation of ginsenoside Rg3 by commercial enzymes. Some commercial enzymes, Cellulase-12T, Protease NP, Viscozyme L, Celluclast 1.5L FG, Lactozym, Pectinex 5XL, Novozym 435, and Cytolase, were added to the white ginseng extract. The results indicated that the hydrolysis of Cellulase-12T was more effective than other enzymes, by hydrolyzing the *β*-glycosidic linkage at C-20 in protopanaxadiol-type ginsenosides. Besides, the content of 20(S)-ginsenoside Rg3 could improve 4 times after treated with Cellulase-12T for 72 h, comparing with commercial white ginseng extract [37]. It is known that aglycone protopanaxatriol is easy to be absorbed and has broader prospect than other saponins. Aglycone protopanaxatriol can be obtained by hydrolyzing the saponins. A recombinant *β*-glycosidase obtained from* Dictyoglomus turgidum* specifically hydrolyzed the xylose at the C-6 position and the glucose in protopanaxatriol- (PPT-) type ginsenosides. The specific activity of* D. turgidumβ*-glycosidase followed the order: Rf > Rg1 > Re > R1 > Rh1 > R2. But* D. turgidumβ*-glycosidase did not have effect on Rg2. So* D. turgidumβ*-glycosidase hydrolyzed the *β*-D-glucopyranose at the C-6 position and C-20 position and cannot hydrolyze *α*-L-rhamnopyranoside at the C-6 position of Rg2. The optimal reaction conditions for APPT production using* D. turgidumβ*-glycosidase were pH 6.0, 80°C. The transformed pathways of PPT-type ginsenosides using* D. turgidumβ*-glycosidase were as follows: R1 → R2 → Rh2 → APPT, Rg1 → Rh1 → APPT, Rf → Rh1 → APPT, Re → Rg2 [38].

### 4.2. Intestinal Bacterial Hydrolysis

Many researchers study the metabolism of ginsenosides in vivo by intestinal microflora and found that the real effective component of ginsenosides is aglycone, which laid the foundation to the development of new drugs. The study found that 20-O-*β*-D-glucopyranosyl-20(S)-protopanaxadiol (IBM I), the intestinal bacterial metabolite of Rb1, was really resistant to metastasis instead of Rb1. And the antimetastatic efficacy of IBM I was better than that of Rb1 and at least comparable to that of 5-FU. And the cure rate of IBM I was greater than amputation alone in a leg amputation [39]. Besides, nine metabolites (M1–M9) of ginsenoside Rb1 were detected in rat feces and eight metabolites (M1, M2, M4–M8, and M10) were detected in rat urine. The structures of ten metabolites were identified as ginsenoside Rd, gypenoside XVII, 20(S)-ginsenoside Rg3, 20(R)-ginsenoside Rg3, ginsenoside F2, compound K, 12-hydroxydammar-3-one-20(S)-O-*β*-D-glucopyranoside, and 25-hydroxyl-(E)-20(22)-ene-ginsenoside Rg3, respectively. It is significant to know that four metabolites (M7–M10) were first reported in vivo. And the highest concentration of deglycosylated metabolites in rat urine and feces appeared during 6–12 h and 24–36 h, respectively [40]. What is more, the same hydrolysates were found in human plasma and urine after human take oral extract but were not found in the extract. This result suggested that products must result from either the degradation or metabolism of ginsenosides in the human body. The major hydrolysates were ginsenoside Rh1, ginsenoside F1, and compound K. And compound K was the main metabolite of the protopanaxadiol ginsenosides [41]. The interesting thing is when ginsenoside Rb1 was given orally to germ-free rat, neither compound K nor any other metabolite was detected in the plasma, intestinal tract, or cumulative feces. In contrast, when ginsenoside Rbl was given orally to gnotobiote rats monoassociated with* Eubacterium* sp. A-44, compound K was found in the plasma, caecum, and cumulative feces. The result indicated that ginsenoside Rb1 was not easily absorbed into body. Nevertheless, compound K, the metabolic of ginsenoside Rb1, was easily absorbed into body. However, not all human intestinal could transform the ginsenoside Rb1.* Eubacterium* sp. A-44 is the only one intestinal strain from man that can convert ginsenoside Rbl into compound K via ginsenoside Rd. The transformation pathway of ginsenoside Rbl by intestinal bacterial was as followed: Rbl → Rd → F2 → C-K → 20(S)-protopanaxadiol [42]. Furthermore, saponins can also be converted into metabolites in anaerobic environment. The possible transform pathways of ginsenosides Rb1, Rb2, Rc, Re, and Rg1 were as follows: Rb1 → Rd → F2 → 20-O-*β*-D-glucopyranosyl-20(S)-protopanaxadiol, Rb2 → unknown-substance → 20-O-[*α*-L-arabinopyranosyl(1 → 6)-*β*-D-glucopyranosyl]-20(S)-protopanaxadiol → 20-O-*β*-D-glucopyranosyl-20(S)-protopanaxadiol, Rc → Rh1 → 20-O-[*α*-L-arabinofuranosyl(1 → 6)—*β*-D—glucopyranosyl]-20(S)-protopanaxadiol → 20-O-*β*-D-glucopyranosyl-20(S)-protopanaxadiol, Re → Rg1 → F1 → 20(S)-protopanaxatriol, Rg1 → F1 → 20(S)-protopanaxatriol [43]. In addition, some human intestinal bacteria could transform ginsenoside Rg3 and △20-ginsenoside Rg3 to ginsenoside Rh2 and △20-ginsenoside Rh2 and protopanaxadiol. For example,* Bacteroides* sp.,* Eubacterium* sp., and* Bifidobacterium* sp. could convert ginsenoside Rg3 to protopanaxadiol via ginsenoside Rh2, but* Fusobacterium* sp. metabolized ginsenoside Rg3 to ginsenoside Rh2 alone [30, 44]. The possible transformation pathway of Rg3 by fusobacterium K-60 was as follows: ginsenoside Rg3 → ginsenoside Rh2 → protopanaxadiol, Rg3 → △20-ginsenoside Rh2 → △20-protopanaxadiol [44]. Another interesting thing is that 20-O-*β*-D-glucopyranosyl-20(S)-protopanaxadiol, a metabolite of ginsenosides, was accumulated into the liver soon after its intravenous administration to mice and its metabolite was fatty acid ester that accumulated in the liver longer than it. Fatty acid ester inhibited tumor growth more than 20-O-*β*-D-glucopyranosyl-20(S)-protopanaxadiol in vivo, which suggested that the real active substance of ginsenosides in the body may be fatty acid ester [30, 45].

### 4.3. Endophytic Bacteria Transformation

Endophyte is the fungus or bacterium that lives in the tissues and organs of healthy plants at a certain stage or all stage. 10 *β*-glucosidase-producing endophytic bacteria were isolated from ginseng roots. Among these endophytic bacteria,* Burkholderia* sp. GE 17-7 has a strong ability to convert the major ginsenoside Rb1 and Rd into minor ginsenoside Rg3 via hydrolyzing the outer glycosidic linkage at C-20 position. The pathway of ginsenoside Rb1 by the strain GE 17-7 was as follows: ginsenoside Rb1 → ginsenoside Rd → ginsenoside Rg3. The optimal conditions were pH 7.0, 30°C, 15 h and the maximum conversion rate of ginsenoside Rg3 was 98% [46]. Ginsenosides Rb1 and Rd were also transformed into gypenoside LXXV and compound K by strain CNU 120806, respectively, by hydrolyzing the terminal and inner glucopyranosyl moieties at the C-3 carbon. The transformation pathways of ginsenosides Rb1 and Rd were as follows: ginsenoside Rb1 → gypenoside XVII → gypenoside LXXV; ginsenoside Rd → F2 → compound K. The optimal conditions for transformation by strain CNU 120806 are 50°C and pH 5.0 [47]. Besides,* Esteya vermicola* CNU 120806 has the high specific activity in converting ginsenoside Rg3 to Rh2. However, ginsenoside Re was not converted by strain CNU 120806. The production rate of Rh2 reached 90.7%. The optimal condition was as follows: 50°C, pH 5.0, and substrate concentration of 3 mg·ml^−1^ [48]. An endophyte JG09 isolated from* Platycodon grandiflorum* also has the ability for converting the ginseng total saponins and ginsenoside monomers. For example, endophyte bacteria JG09 could transform ginsenosides Rb1, Rb2, Rc, and Rd into ginsenosides F2 and compound K and transformed ginsenoside Rg1 into ginsenoside Rh1. The probable pathways of Rb1, Rb2, Rc, and Rg1 by endophyte JG09 were as follows: Rb1 → Rd → F2 → C-K; Rb2 → C-O → C-Y → C-K; Rc → C-Mc1 → C-Mc → C-K; Rg1 → Rh1. (Table 1). And the maximum production rate of ginsenoside F2 and compound K reached 94.53% and 66.34% on the condition of PH 4.0, respectively [49].

### 4.4. Edible Fungi Transformation

Microorganisms applied on the transformation of ginsenoside do not have the food-grade standards. But on the influence of food safety, microorganisms isolated from food have a great prospect at industrial production.* Leuconostoc mesenteroides* DC102 strain and* Lactobacillus pentosus *strain 6105 were isolated from kimchi, a traditional Korean fermented food.* Leuconostoc mesenteroides* DC102 would convert ginsenoside Rb1 to gypenoside XVII, ginsenoside Rd, ginsenoside F2, and compound K.* Leuconostoc mesenteroides* DC102 converted ginsenoside Rb1 to compound K by hydrolyzing the two glucose molecules at C-3 and one of the glucose molecules at C-20, suggesting the conversion pathway: ginsenoside Rb1 → gypenoside XVII and ginsenoside Rd → ginsenoside F2 → compound K. The optimal conditions of conversion were as follows: 30°C, 72 h and PH 6.0 to 8.0. [50, 51].* Lactobacillus paralimentarius* LH4 is also isolated from kimchi and it transformed ginsenoside Rb1 into compound K via gypenoside XVII, ginsenoside Rd, and ginsenoside F2 orderly, through hydrolyzing the two glucose moieties at C-3 and the outer glucose moiety at C-20 position of the ginsenoside Rb1. And the optimal conditions of conversion by* Lactobacillus paralimentarius *LH4 were 30°C, pH 6.0, and 72 h [52]. In addition, some food microorganisms could convert ginsenoside Rb1 and Re into rare ginsenosides. The conversion pathway of ginsenoside Rb1 and Re by food microorganisms can be traced as follows: Rb1 → Rd and F → compound K, Rb1 → Rd and F2 → Rh2, Rb1 → Rd → F2, Re → Rg2 → Rh1, Re → Rg1 → Rh1 [53]. Ginsenosides Rb2 and Rc were also transformed by food microorganisms. The conversion pathways of ginsenosides Rb2 and Rc were as follows: Rb2 and Rc → Rd → F2 → Rh2 or compound K, Rb2 → compound O → compound Y → compound K, Rc → compound Mc → compound K [54] (Table 2).

### 4.5. Soil Microbial Transformation

Soil microbes are the microscopic organisms that are invisible to the naked eye or invisible to the soil. Strictly speaking, they include bacteria, archaea, fungi, viruses, protozoa, and microscopic algae. They can make chemical reactions in the soil and promote the decomposition of soil organic matter and the transformation of nutrients. Twenty-two microorganisms, producing *β*-glucosidase, were isolated from the ginseng filed. Among these microorganisms, GH9, GH21, and GH26 have the ability to transform ginsenoside Rb1 to less polar metabolite. Only GH21 was able to transform ginsenoside Rg1. GH21 was identified as a* Cladosporium cladosporioides* species by comparing rRNA gene sequences. The proposed conversion pathways of ginsenosides Rb1 and Rg1 by GH21 were as follows: Rb1 → Rd or gypenoside XVII → F2 → C-K, Rg1 → F1, respectively. Ginsenosides Rb1 and Rg1 bioconversion rates were 74.2% and 89.3%, respectively [55].* Aspergillus versicolor *strain LFJ1403, producing *β*-glucosidase, is also isolated from ginseng filed. Strain LFJ1403 could transform ginsenoside Rb1 into ginsenoside Rd as the sole product by hydrolyzing the outer glucose moiety at C-20 position. The yield of conversion was up to 96% on the optimal biotransformation conditions, 37°C, PH5.0, and 48 h [56]. In addition, many microorganisms were obtained around the ginseng roots in fields. Among these microorganisms, a strain of fungus GF06 was identified as* Aspergillus niger* and showed a strong ability to transform Rg3(S, R) into PPD(S, R) via the following pathway: Rg3 → Rh2 → PPD, Rg3 → PPD. The conversion rate of Rg3(S, R) into PPD(S, R) reached 100%, and the optimal conditions were PH5.0 and 55°C [57]. What is more,* Aspergillus niger* also could transform ginsenoside Rf to 20(S)-protopanaxatriol by hydrolyzing the terminal glucose and then inner glucose at the C-6 position, suggesting the transformation pathway: Rf → Rh1(S) → PPT(S). The yield of conversion was up to 90.4% on the optimal conditions, PH 5.0, 55°C and substrate concentration 1.25 mmol/l [58]. Beside,* Microbacterium esteraromaticum* would convert ginsenoside Rb2 into compound Y and compound K. The possible conversion mechanism was as follows: Rb2 → compound Y → compound K [59]. Moreover,* Fusarium sacchari, *isolated from the soil-cultivated ginseng, could transform leaves of total saponins into compound K, compound Mx, and ginsenoside Mc, which have the strong pharmacological activity. And the levels of C-K, C-Mx, and G-Mc were the highest in the PDA medium, comparing with martin, wort media, and Zpek medium. The optimal transforming conditions for C-K, C-Mx, and G-Mc by* Fusarium sacchari* were as follows: 30°C, 6 days, and PH 5.5. The content of C-K converted by* Fusarium sacchari* was increased by 215 times those of the control [60] (Table 3).

## 5. Conclusion

In conclusion, the transformation of ginsenosides is achieved mainly by hydrolyzing the glycosidic bond at C-3, C-6, and C-20 position of the ginsenosides. Transformation of saponins by physical methods is not suitable for industrial production because of the high reaction conditions, the huge energy consumption, and the lower level of saponins. For instant, the content of PPD and PPT would be decreased at steaming. The secondary saponins prepared by chemical methods were easy to hydroxylate, hydrolyze, and isomerize. There are some problems in reaction progress, such as violent reaction, uncontrolled hydrolysis degree, difficult purification, and environmental pollution. Moreover, the transformation rate of secondary saponins was not high by chemical methods. Comparing the physical methods and chemical methods, biotransformation has quite a few advantages in the transformation of ginsenosides. For example, biotransformation is a fast, convenient, efficient, and environmental friendly way to obtain rare saponins. Because of these advantages, biotransformation is also the most commonly used method to convert ginsenoside. More significantly, China is the first to realize the industrial production of the Rh2 and other rare saponins by using enzymatic method [61]. In recent years, a large number of researches have been studied for the conversion of major ginsenosides and have achieved great results. However, these methods cannot meet the requirement of industrialization in producing rare ginsenosides. It needs more researchers to explore the way to produce the rare ginsenosides and be applied on large-scale production of saponins.

## Figures and Tables

**Table 1:** Ginsenosides converted by endophytic bacteria.

Ginsenosides	Microorganism	After the transformation	References
Rb1	*Burkholderia *sp.* GE 17-7 *	Rg3	[46]
	*Esteya vermicola CNU 120806*	gypenoside LXXV	[47]
Rb1, Rb2, Rc, Rd	* Luteibacter *sp.	F2, C-K	[49]
Rg1	*Luteibacter *sp.	Rh1	[49]
Rg3	*Esteya vermicola CNU 120806 *	Rh2	[48]

**Table 2:** Ginsenosides converted by edible fungi.

Ginsenosides	Microorganism	After the transformation	References
Rb1	*Leuconostoc mesenteroides DC102 *	Prosapogenins, Rd, F2,	[51]
		gypenoside XVII, C- K	
	*Lactobacillus pentosus strain 6105*	PPD	[52]
	*Lactobacillus paralimentarius,*	C-K	[53, 54]
	*Bifidobacterium *sp.* Int57, Bif. *sp.* SJ32, Aspergillus niger and A. usamii *		
	* Lactobacillus delbrueckii,*	Rh2	[54]
	*Leuconostoc paramesenteroides *		
	*Bifidobacterium *sp.* SH5*	F2	[54]

Re	*Bif. *sp.* Int57, Bif. *sp.* SJ32, A. niger *	Rh1	[54]
	*A. usamii*	Rg2	[54]

Rb2, Rc	*Bifidobacterium *sp.* Int57, *	C-K	[55]
	*Bifidobacterium *sp.* SJ32,*		
	*Bifidobacterium *sp.* SH5*		
	*Lactobacillus delbrueckii *	Rh2	[55]

**Table 3:** Ginsenosides converted by soil microbacteria.

Ginsenosides	*Microorganism*	After the transformation	References
Rb1, Rg1	*Cladosporium cladosporioides*	C–K, F1	[56]
Rb1	*Aspergillus versicolor*	Rd	[57]
Rb2	*Microbacterium esteraromaticum*	C-Y, C-K	[60]
Rg3(S, R)	*Aspergillus niger*	PPD(S, R)	[58]
Rf	*Aspergillus niger*	20(S)-protopanaxatriol	[60]
Rb1, Rb2, Rb3, Rc	*Fusarium sacchari*	C-K, C-Mx, G-Mc	[61]
